# Antibiotic Receipt for Pediatric Telemedicine Visits With Primary Care vs Direct-to-Consumer Vendors

**DOI:** 10.1001/jamanetworkopen.2024.2359

**Published:** 2024-03-14

**Authors:** Samuel R. Wittman, Alejandro Hoberman, Ateev Mehrotra, Lindsay M. Sabik, Jonathan G. Yabes, Kristin N. Ray

**Affiliations:** 1 Department of Pediatrics, University of Pittsburgh School of Medicine, UPMC Children’s Hospital of Pittsburgh, Pittsburgh, Pennsylvania; 2 Department of Health Care Policy, Harvard Medical School, Boston, Massachusetts; 3 Department of Health Policy and Management, University of Pittsburgh School of Public Health, Pittsburgh, Pennsylvania; 4 Department of Medicine, University of Pittsburgh School of Medicine, Pittsburgh, Pennsylvania

## Abstract

**Importance:**

Prior research found that pediatric direct-to-consumer (DTC) telemedicine visits are associated with more antibiotic prescribing than in-person primary care visits. It is unclear whether this difference is associated with modality of care (telemedicine vs in-person) or with the context of telemedicine care (primary care vs not primary care).

**Objective:**

To compare antibiotic management during telemedicine visits with primary care practitioners (PCPs) vs commercial direct-to-consumer (DTC) telemedicine companies for pediatric acute respiratory tract infections (ARTIs).

**Design, Setting, and Participants:**

This retrospective, cross-sectional study of visits for ARTIs by commercially insured children 17 years of age or younger analyzed deidentified medical and pharmacy claims in OptumLabs Data Warehouse data, a national sample of commercial enrollees, between January 1 and December 31, 2022.

**Exposure:**

Setting of telemedicine visit as PCP vs DTC.

**Main Outcomes and Measures:**

The primary outcome was percentage of visits with antibiotic receipt. Secondary outcomes were the percentages of visits with diagnoses for which prescription of an antibiotic was potentially appropriate, guideline-concordant antibiotic management, and follow-up ARTI visits within the ensuing 1 to 2 days and 3 to 14 days. The ARTI telemedicine visits with PCP vs DTC telemedicine companies were matched on child demographic characteristics. Generalized estimated equation log-binomial regression models were used to compute marginal outcomes.

**Results:**

In total, data from 27 686 children (mean [SD] age, 8.9 [5.0] years; 13 893 [50.2%] male) were included in this study. There were 14 202 PCP telemedicine index visits matched to 14 627 DTC telemedicine index visits. The percentage of visits involving receipt of an antibiotic was lower for PCP (28.9% [95% CI, 28.1%-29.7%]) than for DTC (37.2% [95% CI, 36.0%-38.5%]) telemedicine visits. Additionally, fewer PCP telemedicine visits involved receipt of a diagnosis in which the use of antibiotics may be appropriate (19.0% [95% CI, 18.4%-19.7%] vs 28.4% [95% CI, 27.3%-29.6%]), but no differences were observed in receipt of nonguideline-concordant antibiotic management based on a given diagnosis between PCP (20.2% [95% CI, 19.5%-20.9%]) and DTC (20.1% [95% CI, 19.1%-21.0%]) telemedicine visits. Fewer PCP telemedicine visits involved a follow-up visit within the ensuing 1 to 2 days (5.0% [95% CI, 4.7%-5.4%] vs 8.0% [95% CI, 7.3%-8.7%]) and 3 to 14 days (8.2% [95% CI, 7.8%-8.7%] vs 9.6% [95% CI, 8.8%-10.3%]).

**Conclusions and Relevance:**

Compared with virtual-only DTC telemedicine companies, telemedicine integrated within primary care was associated with lower rates of antibiotic receipt and follow-up care. Supporting use of telemedicine integrated within pediatric primary care may be one strategy to reduce antibiotic receipt through telemedicine visits.

## Key Points

**Question:**

How does antibiotic management for pediatric acute respiratory tract infection (ARTI) during telemedicine visits with primary care practitioners (PCPs) compare with visits with commercial direct-to-consumer (DTC) companies?

**Findings:**

This cross-sectional study of 27 686 children with ARTI visits delivered virtually found that PCP telemedicine visits were less likely than virtual-only DTC telemedicine visits to involve receipt of antibiotics, receipt of a diagnosis that warranted antibiotics, or a follow-up visit or antibiotic fill during the subsequent 2 weeks. The PCP and DTC telemedicine visits had similar rates of antibiotic management that were consistent with the stated diagnosis.

**Meaning:**

These findings suggest that ARTI antibiotic management via telemedicine differed by context of telemedicine use such that telemedicine policy should account for different models and quality of telemedicine care and support integration of telemedicine within pediatric primary care.

## Teaser

This cross-sectional study compares antibiotic management during telemedicine visits with primary care practitioners vs with commercial direct-to-consumer telemedicine companies for treatment of acute respiratory tract infection in children.

## Introduction

Prior to the COVID-19 pandemic, commercial direct-to-consumer (DTC) telemedicine was the form of telemedicine most commonly used for children.^1,2^ Direct-to-consumer telemedicine is virtual-only care staffed by clinicians not part of the patient’s primary care practice, offering care on demand to address common acute concerns, such as acute respiratory tract infections (ARTIs). Acute respiratory tract infections include a cluster of viral and bacterial infections, such as acute otitis media, sinusitis, streptococcal pharyngitis, and viral upper respiratory tract infections, which make up 20%-50% of acute pediatric visits across different settings.^2,3,4^ Prior research showed substantially higher antibiotic prescribing to children during DTC telemedicine visits compared with in-person visits provided by primary care practitioners (PCPs).^5^ Those results left unclear whether this difference was associated with modality of care (telemedicine vs in-person) or context of telemedicine care (eg, primary care vs not primary care).

Modality of care (telemedicine vs in-person care) could be associated with these observed differences. Telemedicine limits some examination maneuvers (eg, palpation), requires specific ancillary devices to perform others (eg, tele-otoscopy), and requires additional processes or travel to facilitate diagnostic testing (eg, streptococcal testing).^6^ Additionally, virtual connections may be dropped or of low quality, potentially reducing the quality of communication.^7^ Alternatively, context (primary care vs not primary care) could be the more influential factor in prior studies due to differences in clinicians and in continuity. For example, PCPs caring for children are generally pediatricians or family practitioners who have training and experience in in-person pediatric care, while virtual-only practitioners may not have pediatric training or experience caring for children in-person. Additionally, PCPs enter an encounter with prior records, ongoing relationships, and the ability to coordinate in-person follow-up.^8^ Supporting the idea that these contexts may matter more than modality, 1 prior study^9^ found that when the same emergency clinicians practiced in both in-person and telemedicine modalities, their antibiotic prescribing rates were similar in both settings. Before the COVID-19 pandemic, there was low PCP telemedicine use, making it difficult to directly evaluate whether outcomes differ for telemedicine with PCPs compared with DTC telemedicine.

During the first 18 months of the pandemic, the vast majority of pediatric telemedicine visits were provided by PCPs, as opposed to DTC telemedicine.^3^ In 1 study^10^ during the first year of the COVID-19 pandemic, antibiotic prescribing at PCP telemedicine visits was lower than PCP in-person visits, with high guideline concordance through both modalities. These findings provide support again to the idea that context rather than technology itself may be a key factor in prior findings. Throughout the subsequent years, rates of telehealth use, viral epidemiology, and antibiotic use have continued to evolve.^4,11,12,13^ To better understand antibiotic management in different telemedicine contexts in the current health care landscape, direct comparisons of PCP telemedicine and DTC telemedicine visits are needed.

Thus, to fill this knowledge gap, we used 2022 national commercial health plan data to compare matched PCP telemedicine and DTC telemedicine ARTI visits on antibiotic receipt, diagnoses received, guideline-concordant antibiotic management, and follow-up visits. Comparing outcomes for these 2 settings can inform post–public health emergency telemedicine payment and regulatory policy to support telemedicine use that facilitates high-quality care for children.

## Methods

### Study Population

In this cross-sectional analysis, we examined visits of commercially insured children 0 to 17 years of age occurring between January 1, 2022, and December 31, 2022. Data came from the OptumLabs Data Warehouse, which contains deidentified medical and pharmacy claims from a national sample of commercial enrollees. By focusing on 2022, we examined care during a time when PCPs had 2 prior years of telemedicine experience and when in-person visits and ARTI visit rates had rebounded after an early pandemic lull.^4^ This analysis of deidentified data was determined by the Harvard Medical School Institutional Review Board to be exempt from review and the requirement for informed consent because it was not considered human participants research. This study followed the Strengthening the Reporting of Observational Studies in Epidemiology (STROBE) reporting guideline for cross-sectional studies.

### Identifying ARTI Index Visits

**Figure 1.**
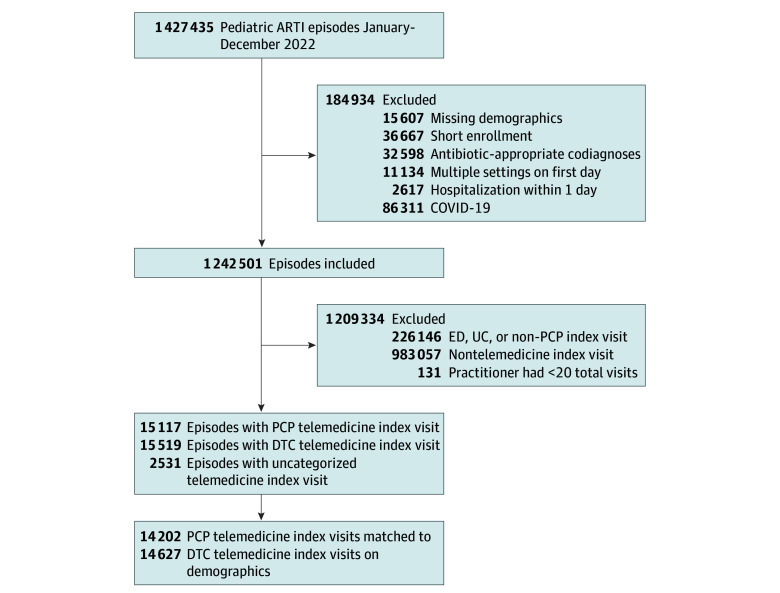
Flow Diagram of Included Pediatric Acute Respiratory Tract Infection (ARTI) Episodes With Initial Visit Through Either Primary Care Practitioner (PCP) Telemedicine or Direct-to-Consumer (DTC) Telemedicine ED indicates emergency department; UC, urgent care.

We identified ARTI visits using previously described methods based on *International Statistical Classification of Diseases, Tenth Revision* (*ICD-10*) codes for streptococcal pharyngitis, acute otitis media, sinusitis, influenza, bronchiolitis or bronchitis, and viral upper respiratory infection (URI) (eTable 1 in Supplement 1).^14,15,16^ We constructed episodes of care that included index ARTI visits (defined as no ARTI encounter in the prior 21 days) and any related follow-up visits (occurring within 14 days of an index visit) to facilitate analysis based on where the first (or index) visit occurred. After constructing episodes, we excluded episodes with less than 1 month of medical and pharmaceutical coverage or lacking demographic data required for matching (4% of episodes) (Figure 1; eMethods in Supplement 1), codiagnoses potentially altering antibiotic prescribing (2% of episodes),^14^ multiple visits on the first day (1% of episodes), hospitalization within 1 day (0.2% of episodes), or a COVID-19 diagnosis at the index visit (6% of episodes; due to antibiotic prescribing described elsewhere for these visits).^16^

### Categorizing ARTI Index Visits

We categorized episodes based on index visit modality (telemedicine vs in-person (eMethods in Supplement 1) and site (eg, PCP, emergency department) using *Current Procedural Terminology* codes and modifiers, place-of-service codes, provider specialty codes, and revenue codes.^15,16^ We excluded episodes with index visits that were not via telemedicine and visits to emergency departments, urgent care, or other nonprimary care sites. Each provider identifier for telemedicine visits was categorized based on the volume and proportion of all visits billed under that provider identifier that were conducted via telemedicine (including visits to all ages and for all diagnoses). We recognize that these provider identifier codes could represent a clinic, health system, DTC telemedicine company, or individual clinician. We excluded provider identifiers with fewer than 20 total visits in the claims, due to concerns about the accuracy of telemedicine proportion with few claims.

Of the remaining provider identifiers, we identified those having fewer than 50% of visits conducted via telemedicine. These visits were categorized as PCP telemedicine (eTable 2 in Supplement 1). Among provider identifiers with more than 80% of visits conducted via telemedicine, we identified a distinct cluster of 6 provider identifier codes that had volume of more than 10 000 total visits in 2022, of which greater than 97% were classified as telemedicine. We categorized these provider identifiers as DTC telemedicine. Less than 8% of ARTI telemedicine index visits were billed under a provider identifier code that did not fall into 1 of these 2 classifications; these were excluded from our main analysis (351 provider identifiers, 2531 index ARTI visits).

### Primary Outcome

Our primary outcome was whether the index visit resulted in an antibiotic fill. We identified systemic antibiotic fills in pharmacy claims using American Hospital Formulary Service Pharmacologic-Therapeutic Classification System codes.^17^ If an antibiotic fill occurred on the same day or the ensuing 2 days as an ARTI visit, that antibiotic fill was linked with the visit. If an antibiotic fill could potentially be associated with multiple ARTI visits, we assigned the antibiotic fill to the visit most immediately before the fill.

### Secondary Outcomes

In our main analysis, secondary outcomes focused on 3 additional aspects of care: diagnosis received during the index visit, guideline-concordance of antibiotic management during the index visit based on diagnosis received; and follow-up care received after the index visit. Examining the diagnosis received along with the guideline concordance of antibiotic management conditional on diagnosis enabled us to separately examine 2 linked components that may be associated with antibiotic overuse: overdiagnosis and overtreatment. Overdiagnosis of bacterial conditions and unnecessary prescribing for viral illnesses may both contribute to excess antibiotic use.^18^ Examining follow-up care (including follow-up ARTI visits and antibiotics) enabled us to potentially capture the sequelae of underdiagnosis or misdiagnosis, which could lead to a need for revisits or alternative management.

As already noted, we examined the diagnosis received as an outcome because overdiagnosis of bacterial conditions is 1 mechanism that may contribute to antibiotic overuse. We recognize the possibility of true differences in case mix between sites (ie, symptoms or symptom severity may influence choice of PCP telemedicine vs DTC telemedicine), but we are also cognizant of the subjectivity in diagnosis and the potential for overdiagnoses of bacterial infections as a means of justifying an antibiotic prescription.^19^ Using *ICD-10* codes (eTable 1 in Supplement 1), we categorized each index visit into 1 of 6 primary diagnoses groups. If multiple diagnoses were present, visits were categorized as streptococcal pharyngitis, acute otitis media, or sinusitis if 1 of these “antibiotics may be indicated” diagnoses was present, and as influenza, bronchiolitis or bronchitis, or viral URI only if none of the prior “antibiotics may be indicated” diagnoses were present, consistent with prior studies.^5^

Next, we examined guideline concordance of antibiotic management based on assigned diagnosis using guidelines’ recommendations.^20,21,22,23^ This measure examined antibiotic management for each visit in the context of the stated diagnosis, and so complements the prior measure of diagnosis. We considered antibiotic management to be guideline-concordant if a patient with streptococcal pharyngitis received penicillin or amoxicillin; if a patient with acute otitis media or bacterial sinusitis received amoxicillin or amoxicillin/clavulanic acid or no antibiotic; or if a patient with a viral ARTI diagnosis received no antibiotic (eTable 3 in Supplement 1). Nonconcordance could occur in 3 ways: lack of antibiotics when indicated; use of a nonguideline-concordant antibiotic when a guideline-concordant antibiotic was indicated; and use of an antibiotic when not indicated.

Finally, we examined follow-up care after index ARTI visits as a way to assess for potential misdiagnosis or undertreatment at the index visit. As described in the eMethods in Supplement 1, visits with ARTI diagnoses that occurred within 14 days of an index visit were linked to the index visit as follow-up ARTI visits. Antibiotic fills occurring in the same day or within the subsequent 2 days of these ARTI follow-up visits were identified as follow-up ARTI antibiotic fills. As an alternative more sensitive measure, we also examined any antibiotic fill within 14 days of the index visit but not associated with the index visit itself to capture more broadly any antibiotics prescribed outside of a billed encounter (eg, phone or patient portal communication) or with a new, alternative diagnosis (eMethods in Supplement 1).

### Matching

We used exact matching for PCP telemedicine index visits and DTC telemedicine index visits.^24,25^ Visits were matched on the following 6 sociodemographic variables: child sex, age group (0-1, 2-5, 6-11, and 12-17 years), medical complexity status (categorized using the pediatric medical complexity algorithm as no chronic conditions, noncomplex chronic conditions, or complex chronic conditions^26^), state of residence, and urban-rural status based on the rural-urban commuting area code (metropolitan, micropolitan, small town, and rural).^27^ Observations were matched many-to-many, pruned, and weighted for balance of covariates. Weights were generated for each matched set (with each set treated as a stratum) to align distributions of matched covariates for DTC telemedicine visits with PCP telemedicine index visits. All analyses of matched visits include application of these weights.

### Statistical Analysis

We first compared the frequency distribution of covariates in episodes with PCP telemedicine index visits vs DTC telemedicine index visits before and after matching. Next, we compared our primary outcome (index visit antibiotic receipt) and secondary outcomes (diagnosis received, guideline-concordant antibiotic management, and ARTI follow-up visits and antibiotics) through separate regressions. To account for clustering of visits within each child, we computed marginal (population-averaged) outcome proportions using generalized estimating equation log-binomial regression models with telemedicine site as the independent variable. An independent within-child correlation structure was used based on the quasi-likelihood under the independence model criterion. Models used robust standard errors for inference. Predictive marginal proportions and relative risks (RR) comparing PCP telemedicine against DTC telemedicine with 95% CIs were calculated from the models. We determined whether a difference between sites was statistically significant using 95% CI for RR. Statistical significance was defined as a 95% CI excluding 1. We further contextualized results with additional post hoc analyses (eg, factors associated with increased nonconcordant antibiotic use, sites of follow-up care, rural vs urban residence of child, visits without audio-only *Current Procedural Terminology* codes, presence of any *ICD-10* code indicating antibiotic allergy [Z88, T78] from 2020 through 2022). Analyses were conducted in SAS, version 9.4 (SAS Institute Inc ). In 2 sensitivity analyses, we examined robustness of findings to assumptions about the potential counterfactual diagnosis that patients may have received had they presented elsewhere. In our first sensitivity analysis, we treated diagnosis as more objective than in the main analysis by assuming that diagnosis for a given child would not vary with alternative site of presentation, and therefore, we matched visits on diagnosis in addition to sociodemographic characteristics. In our second sensitivity analysis, we allowed for greater subjectivity in diagnosis than the main analysis by including visits that did not have ARTI diagnoses but did have ARTI symptom *ICD-10* codes (eg, cough or rhinorrhea) (eTable 1 in Supplement 1) during our construction of episodes of ARTI care, thereby assuming that a child receiving a cough diagnosis at one site could have received a “viral URI” diagnosis at another site. Thus, these 2 sensitivity analyses examined the degree to which our findings varied with more rigid vs relaxed assumptions about the diagnoses that may have been received had the child presented to another site of care.

We also performed 2 additional sensitivity analyses to interrogate the robustness of our results to alternative ways of categorizing the visits occurring with telemedicine practitioners that were excluded in the main analysis (ie, including them with either the DTC or PCP telemedicine categories). After building these alternative sets of matched index visits and weighting, we repeated the analysis described previously, and present RR obtained through each of these analyses.

## Results

### Sample and Matching

Data from 27 686 children (mean [SD] age, 8.9 [5.0] years; 13 793 [49.8%] female; and 13 893 [50.2%] male) were included in this study. Prior to matching, the sample included 15 117 ARTI episodes with PCP telemedicine index visits and 15 519 ARTI episodes with DTC telemedicine index visits. In the unmatched sample, the PCP telemedicine index visits contained higher proportions of visits with younger children, with children with chronic conditions, and with children from Northeast and West census regions compared with DTC telemedicine index visits (Table 1). A total of 14 202 PCP telemedicine index visits were matched on child sociodemographic characteristics to 14 627 DTC telemedicine index visits, resulting in balanced sociodemographic covariates in the matched, weighted sample (Table 1).

**Table 1.** Participant Demographics for Unmatched and Matched Index Telemedicine Visits in the Main Analysis

Characteristic	Unmatched visits, No. (%)		Matched visits, weighted %	
	PCP telemedicine	DTC telemedicine	PCP telemedicine	DTC telemedicine
No. of unweighted visits	15 117	15 519	14 202	14 627
Child age range, y				
0-1	1471 (10)	527 (3)	9	9
2-5	3990 (26)	3325 (21)	26	26
6-11	4804 (32)	5921 (28)	32	32
12-17	4852 (32)	5746 (37)	33	33
Child sex				
Male	7611 (50)	7749 (50)	50	50
Female	7501 (50)	7758 (50)	50	50
Unknown	<11 (<0.1)	12 (<0.1)	0	0
Medical complexity				
No chronic condition	10 022 (66)	11 086 (71)	68	68
Noncomplex chronic condition	3397 (22)	3112 (20)	22	22
Complex chronic condition	1698 (11)	1321 (9)	10	10
US census region				
Northeast	1649 (11)	713 (5)	10	10
South	7115 (47)	9052 (58)	48	48
Midwest	2702 (18)	2896 (19)	18	18
West	3651 (24)	2858 (18)	24	24
Rural or urban location				
Metropolitan	13 602 (90)	14 011 (90)	93	93
Micropolitan	788 (5)	862 (6)	4	4
Small town	493 (3)	424 (3)	2	2
Rural	234 (2)	222 (1)	1	1

Abbreviations: DTC, direct to consumer; PCP, primary care practitioner.

### Main Analysis

The PCP telemedicine index visits were less likely to result in receipt of antibiotics (28.9% [95% CI, 28.1%-29.7%] of PCP telemedicine visits) compared with DTC telemedicine index visits (37.2% [95% CI, 36.0%-38.5%]) (RR, 0.78 [95% CI, 0.74-0.81]) (Table 2). The PCP telemedicine index visits were less likely to result in a diagnosis in which antibiotics may be appropriate (19.0% of PCP telemedicine visits) compared with DTC telemedicine index visits (28.4%; RR, 0.67 [95% CI, 0.63-0.71]). Specifically, PCP telemedicine visits were less likely to result in a sinusitis diagnosis (9.9% vs 15.5% of DTC telemedicine visits; RR, 0.64 [95% CI, 0.59-0.69]) or an acute otitis media diagnosis (4.0% vs 6.9% of DTC telemedicine visits; RR, 0.59 [95% CI, 0.51-0.59]). The PCP telemedicine visits remained less likely to receive antibiotics and to receive a diagnosis in which an antibiotic may be indicated in analyses stratified by metropolitan vs nonmetropolitan residence (eTable 4 in Supplement 1). Excluding visits with the presence of audio-only telemedicine codes did not substantially alter results (eTable 5 in Supplement 1).

**Table 2.** Outcomes of ARTI Telemedicine Visits, Main Analysis^a^

Visit or Outcome	Weighted % (95% CI)		RR (95% CI)
	PCP telemedicine	DTC telemedicine	
Matched index visits, No.	14 202	14 627	
Index visit			
Received antibiotics	28.9 (28.1-29.7)	37.2 (36.0-38.5)	0.78 (0.74-0.81)
Received diagnosis for which antibiotic may be indicated	19.0 (18.4-19.7)	28.4 (27.3-29.6)	0.67 (0.63-0.71)
Streptococcal pharyngitis	5.1 (4.7-5.5)	6.1 (5.5-6.6)	0.84 (0.75-0.94)
Acute otitis media	4.0 (3.7-4.4)	6.9 (6.0-7.7)	0.59 (0.51-0.69)
Sinusitis	9.9 (9.4-10.4)	15.5 (14.7-16.4)	0.64 (0.59-0.69)
Received diagnosis for which antibiotic is not indicated	81.0 (80.3-81.6)	71.6 (70.4-72.7)	1.13 (1.11-1.15)
Influenza	5.1 (4.7-5.4)	3.3 (2.9-3.8)	1.53 (1.32-1.78)
Bronchitis or bronchiolitis	4.0 (3.7-4.4)	3.5 (3.1-4.0)	1.13 (0.97-1.32)
Other viral	71.9 (71.1-72.6)	64.7 (63.5-65.9)	1.11 (1.09-1.14)
Received antibiotic management not concordant with given diagnosis	20.2 (19.5-20.9)	20.1 (19.1-21.0)	1.01 (0.95-1.07)
Antibiotic indicated but not prescribed	1.0 (0.8-1.2)	0.8 (0.6-1.0)	1.23 (0.94-1.61)
Antibiotic selection not guideline concordant	5.6 (5.2-6.0)	4.8 (4.3-5.3)	1.17 (1.03-1.32)
Antibiotic not indicated but prescribed	13.6 (13.0-14.2)	14.5 (13.7-15.3)	0.94 (0.88-1.01)
Follow-up care within 1-2 d			
Follow-up ARTI visit within 1-2 d	5.0 (4.7-5.4)	8.0 (7.3-8.7)	0.63 (0.56-0.70)
Antibiotics filled after ARTI visit within 1-2 d	1.7 (1.5-1.9)	3.2 (2.7-3.6)	0.53 (0.44-0.64)
Follow-up care within 3-14 d			
Follow-up ARTI visit within 3-14 d	8.2 (7.8-8.7)	9.6 (8.8-10.3)	0.85 (0.78-0.95)
Antibiotics filled after ARTI visit within 3-14 d	3.1 (2.8-3.4)	4.8 (4.2-5.3)	0.65 (0.56-0.75)

Abbreviations: ARTI, acute respiratory tract infection; DTC, direct to consumer; PCP, primary care practitioner; RR, relative risk.

^a^ Results of main analysis in which visits were matched for sociodemographic characteristics.

Accounting for diagnosis, rates of nonguideline-concordant antibiotic management were similar for PCP telemedicine and DTC telemedicine index visits (20.2% vs 20.1%; RR, 1.01 [95% CI, 0.95-1.07]). Among PCP telemedicine visits, nonguideline concordance was lower for visits completed by pediatricians (13.6% [95% CI, 12.7%-14.4%]) and higher for visits completed by family practitioners (25.4% [95% CI, 23.3%-27.5%]); individual practitioner specialty was not available for DTC telemedicine visits. The PCP telemedicine visits were more likely than DTC telemedicine visits to use a nonconcordant antibiotic (5.6% vs 4.8%; RR, 1.17 [95% CI, 1.03-1.32]), but had similar rates of providing an antibiotic when not indicated and of not providing an antibiotic when indicated. Nonguideline-concordant antibiotics most commonly used at both sites were azithromycin (46% of nonguideline concordant antibiotics) and cefdinir (34%). For both sites, use of a nonconcordant antibiotic was higher in December 2022 (7.8% and 7.3%) than the remainder of the year, coinciding with an amoxicillin shortage in the US.^28^ Prior diagnosis codes indicating an antibiotic allergy were found in 7.8% of PCP telemedicine visits and 7.6% of DTC telemedicine visits receiving a nonguideline concordant antibiotic.

The PCP telemedicine index visits had lower rates of follow-up ARTI visits within the ensuing 1 to 2 days (5.0%) compared with DTC telemedicine visits (8.0%; RR, 0.63 [95% CI, 0.56-0.70]). For both sites, when these follow-up visits did occur, they occurred primarily as in-person PCP visits (72% of follow-up visits after DTC and PCP telemedicine). Rates of additional ARTI visits between days 3 and 14 after the index visits were also lower for PCP telemedicine index visits (8.2%) compared with DTC telemedicine index visits (9.6%; RR, 0.85 [95% CI, 0.78-0.95]).

Consistent with lower revisit rates, PCP telemedicine also had lower receipt of antibiotics associated with an ARTI revisit within intervals of both 1 to 2 days (1.7% vs 3.2% for DTC telemedicine visits; RR, 0.53 [95% CI, 0.44-0.64]) and 3 to 14 days (3.1% vs 4.8% for DTC telemedicine visits; RR, 0.65 [95% CI, 0.56-0.75]). With our more sensitive specification of after–index visit antibiotics in which we captured any antibiotics within 14 days (excluding fills associated with the index visit), PCP telemedicine had lower receipt of any antibiotic (7.2% vs 9.8%; RR, 0.73 [95% CI, 0.67-0.81]).

### Sensitivity Analyses

**Figure 2.**
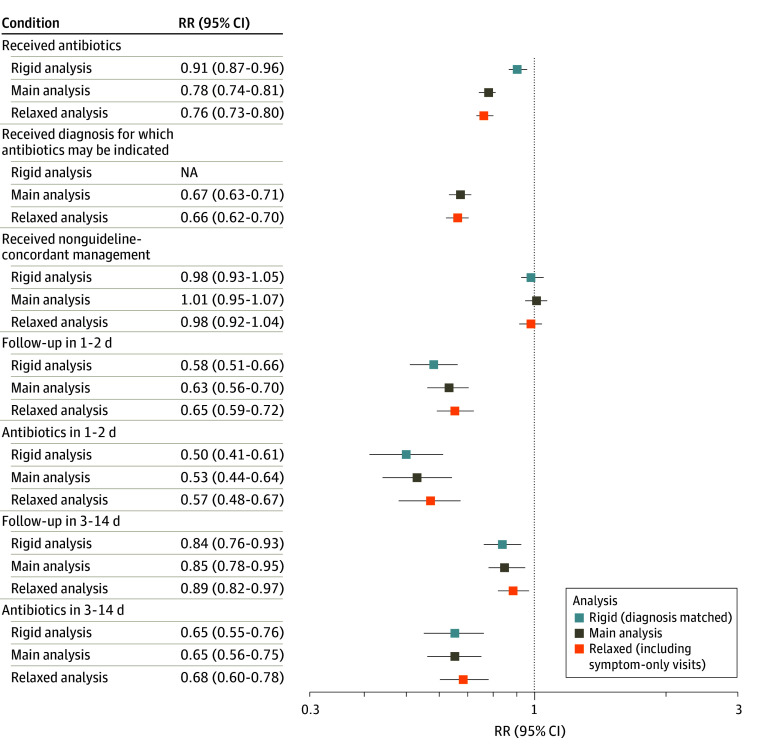
Comparison of Relative Risks (RR) Obtained Through Main Analysis and Sensitivity Analyses Comparison of RRs of select outcomes for primary care practitioner telemedicine visits vs direct-to-consumer telemedicine visits across the main analysis, rigid sensitivity analysis (with visits matched on diagnosis in addition to sociodemographics), and relaxed sensitivity analysis (with visits only matched on sociodemographics and with inclusion of symptom-only visits). NA represents not applicable.

Sensitivity analyses both matching on diagnosis and incorporating visits with diagnoses limited to ARTI symptoms in episode construction did not significantly change our estimates of the association of PCP vs DTC telemedicine with outcomes (eResults, and eTables 6, 7, and 8 in Supplement 1). Across our main and sensitivity analyses, we observed no significant difference in nonguideline concordant care by site; the remaining outcomes occurred less frequently at PCP visits than DTC visits in all analyses (Figure 2) (eResults in Supplement 1). Similarly, after categorizing the practitioners who were excluded in the main analysis within either the DTC telemedicine (eTable 9 in Supplement 1) or PCP telemedicine category (eTable 10 in Supplement 1), we observed estimates of the association of PCP vs DTC telemedicine with outcomes similar to our main analysis.

## Discussion

This cross-sectional analysis found that compared with pediatric ARTI telemedicine visits delivered via virtual-only DTC telemedicine, PCP telemedicine visits were less likely to involve receipt of antibiotics, receipt of a diagnosis that warranted antibiotics, and a follow-up visit or antibiotic fill during the subsequent 2 weeks. The PCP and DTC telemedicine visits had similar rates of providing antibiotic management consistent with the stated diagnosis.

While prior research has reported increased antibiotic use in pediatric DTC telemedicine visits, it has been unclear whether the higher rate of antibiotic receipt observed with DTC telemedicine visits was associated more with the modality of care (telemedicine vs in-person) or context of telemedicine care (eg, telemedicine used in virtual-only, nonprimary care contexts). Our findings support that the difference is largely associated with the context of telemedicine use (rather than telemedicine itself) given the lower rates of antibiotic receipt for PCP telemedicine visits. We found more specifically that DTC telemedicine visits were more likely to result in a diagnosis for which antibiotics could be indicated but a similar rate of guideline-concordant antibiotic management. It is possible that there was a true difference in the case mix of ARTI conditions that presented to each site (perhaps due to differential parent site selection or practice site triage protocols). Alternatively, prior studies indicate that urgent care clinicians with higher rates of antibiotic prescribing are more likely to provide diagnoses for which antibiotics may be indicated and to prescribe antibiotics,^19,29^ highlighting potential subjectivity in diagnoses.

Factors that may be associated with increased diagnosis of bacterial conditions and increased antibiotic prescribing may include increased perception of parent expectations of antibiotics,^30^ clinician incentives emphasizing throughput or patient satisfaction,^18,31,32^ or lack of protocols to transition to in-person care or ensure timely follow-up.^33^ Each of these factors may be more prominent in DTC telemedicine models in which prior relationships are lacking, the ability to follow-up is limited, and clinician incentives may relate to patient ratings or volume. If more nonpediatricians are caring for children in DTC telemedicine than in PCP telemedicine, that may also be associated with these findings, especially since we found that pediatricians and family physicians had different rates of nonguideline-concordant care even within PCP telemedicine visits. While some of these factors, if found to be contributing, may require solutions specific to telemedicine (eg, developing protocols to smoothly transition to local in-person care), other factors may benefit from outpatient antibiotic stewardship methods. Effective interventions to improve existing ARTI quality metrics exist for in-person outpatient settings, and important work is adapting these interventions for telemedicine settings.^14,34,35,36,37,38^ Our results also highlight the need to use antibiotic quality metrics that are diagnosis-specific^39,40^ (which may mask increased antibiotic use due to overdiagnosis) alongside broader measures^41,42^ to monitor and guide improvement in antibiotic management.

Our data suggest that policy in the post–public health emergency era needs to account for the variation within telemedicine models (virtual-only vs integrated) and avoid regulatory and payment strategies that may favor virtual-only DTC telemedicine companies relative to primary care integrated telemedicine care. Our results illustrate that the context of telemedicine care is important for quality; context also influences the feasibility of maintaining telemedicine infrastructure. For example, some decision-makers argue that virtual visits require less overhead costs than in-person care, which is true for virtual-only vendors but not the case for primary care models offering both in-person and virtual care, which must maintain personnel, space, and triage processes for both types of care.^43^ Decreased payments for virtual care relative to in-person care may limit the future of integrated telemedicine models by leading primary care practices to avoid investing in telemedicine at all or to feel incentivized to focus on in-person care rather than virtual care when triaging individual visits.^44^ If primary care practices cannot or choose not to maintain telemedicine infrastructure, families interested in telemedicine turning to virtual-only sites may experience fragmentation and lower quality of care. Policy strategies such as payment parity in fee-for-service models, incentives (financial or otherwise) to primary care practices maintaining telemedicine visits,^45^ or value-based payment models^46^ may help primary care practices to continue to offer telemedicine integrated within their practices.

### Limitations

This study has limitations. As this was an observational analysis of claims data, we lacked clinical information to confirm diagnoses, and therefore performed our primary analysis agnostic to diagnoses and sensitivity analysis specific to diagnoses. We recognize the importance of analyses focused on equity for patients of different racial and ethnic backgrounds; however, patient race, ethnicity, and language information were not available. Future analyses with alternative data should focus on additional patient and practitioner characteristics associated with these outcomes. As this was an observational study, the use of PCP telemedicine and DTC telemedicine was not randomized. While we sought to account for biases using matching, we cannot account for unmeasured variables.

## Conclusions

The results of this cross-sectional study suggest that compared with DTC telemedicine platforms, telemedicine integrated within primary care may perform better in terms of less antibiotic use and reduction of subsequent health care visits. Based on these results, it appears that evolving telemedicine policy may need to account for different models and quality of telemedicine care and should support ongoing integration of telemedicine within pediatric primary care.

## Notes

Supplement 1.
eMethods.

eResults.
**eTable 1.** ICD-10 codes for diagnosis categories **eTable 2.** Visit counts for provider identifier codes identified as PCP telemedicine, DTC telemedicine, and excluded telemedicine providers **eTable 3.** Guideline-concordant antibiotic management by ARTI diagnosis **eTable 4.** ARTI visits and outcomes stratified by non-metropolitan and metropolitan child residence **eTable 5.** ARTI visits and outcomes, excluding visits with the presence of audio-only telemedicine codes **eTable 6.** Demographics and diagnoses of unmatched and matched index visits in sensitivity analysis **eTable 7.** ARTI visits and outcomes, “rigid” sensitivity analysis with index visits matched on diagnosis **eTable 8.** ARTI visits and outcomes, “relaxed” sensitivity analysis including symptom-only visits **eTable 9.** ARTI visits and outcomes, sensitivity analysis assigning visits with “excluded providers” to the DTC telemedicine group **eTable 10.** ARTI visits and outcomes, sensitivity analysis assigning visits with “excluded providers” to the PCP telemedicine group

Supplement 2.
Data Sharing Statement

## References

[1] Barnett ML, Ray KN, Souza J, Mehrotra A. Trends in telemedicine use in a large commercially insured population, 2005-2017. JAMA. 2018;320(20):2147-2149. doi:10.1001/jama.2018.12354 30480716 PMC6349464

[2] Ray KN, Shi Z, Poon SJ, Uscher-Pines L, Mehrotra A. Use of commercial direct-to-consumer telemedicine by children. Acad Pediatr. 2019;19(6):665-669. doi:10.1016/j.acap.2018.11.016 30639759 PMC6620157

[3] Ray KN, Wittman SR, Yabes JG, Sabik LM, Hoberman A, Mehrotra A. telemedicine visits to children during the pandemic: practice-based telemedicine versus telemedicine-only providers. Acad Pediatr. 2023;23(2):265-270. doi:10.1016/j.acap.2022.05.010 35589062 PMC9666718

[4] Schweiberger K, Patel SY, Mehrotra A, Ray KN. Trends in pediatric primary care visits during the coronavirus disease of 2019 pandemic. Acad Pediatr. 2021;21(8):1426-1433. doi:10.1016/j.acap.2021.04.031 33984496 PMC8561008

[5] Ray KN, Shi Z, Gidengil CA, Poon SJ, Uscher-Pines L, Mehrotra A. Antibiotic prescribing during pediatric direct-to-consumer telemedicine visits. Pediatrics. 2019;143(5):e20182491. doi:10.1542/peds.2018-2491 30962253 PMC6565339

[6] McSwain SD, Bernard J, Burke BL Jr, . American Telemedicine Association operating procedures for pediatric telehealth. Telemed J E Health. 2017;23(9):699-706. doi:10.1089/tmj.2017.0176 28829680

[7] Kodjebacheva GD, Culinski T, Kawser B, Coffer K. Satisfaction with telehealth services compared with nontelehealth services among pediatric patients and their caregivers: systematic review of the literature. JMIR Pediatr Parent. 2023;6:e41554. doi:10.2196/41554 37000504 PMC10176140

[8] Conners GP, Kressly SJ, Perrin JM, Richerson JE, Sankrithi UM; Committee on Practice and Ambulatory Medicine; Committee on Pediatric Emergency Medicine; Section on Telehealth Care; Section on Emergency Medicine; Subcommittee on Urgent Care; Task Force on Pediatric Practice Change. Nonemergency acute care: when it’s not the medical home. Pediatrics. 2017;139(5):e20170629. doi:10.1542/peds.2017-0629 28557775

[9] Yao P, Clark S, Gogia K, Hafeez B, Hsu H, Greenwald P. Antibiotic prescribing practices: is there a difference between patients seen by telemedicine versus those seen in-person? Telemed J E Health. 2020;26(1):107-109. doi:10.1089/tmj.2018.0250 30762493

[10] Ray KN, Martin JM, Wolfson D, . Antibiotic prescribing for acute respiratory tract infections during telemedicine visits within a pediatric primary care network. Acad Pediatr. 2021;21(7):1239-1243. doi:10.1016/j.acap.2021.03.008 33741531

[11] Dutcher L, Li Y, Lee G, Grundmeier R, Hamilton KW, Gerber JS. COVID-19 and antibiotic prescribing in pediatric primary care. Pediatrics. 2022;149(2):e2021053079. doi:10.1542/peds.2021-053079 35102416 PMC9825803

[12] Sprecher E, Conroy K, Krupa J, . A mixed-methods assessment of coronavirus disease of 2019-era telehealth acute care visits in the medical home. J Pediatr. 2023;255:121-127.e2. doi:10.1016/j.jpeds.2022.10.036 36372098 PMC9650264

[13] Chua KP, Volerman A, Conti RM. Prescription drug dispensing to US children during the COVID-19 pandemic. Pediatrics. 2021;148(2):e2021049972. doi:10.1542/peds.2021-049972 34285080 PMC8344340

[14] Kronman MP, Gerber JS, Grundmeier RW, . Reducing antibiotic prescribing in primary care for respiratory illness. Pediatrics. 2020;146(3):e20200038. doi:10.1542/peds.2020-0038 32747473 PMC7461202

[15] Wittman SR, Yabes JG, Sabik LM, Kahn JM, Ray KN. Patient and family factors associated with use of telemedicine visits for pediatric acute respiratory tract infections, 2018-2019. Telemed J E Health. 2023;29(1):127-136. doi:10.1089/tmj.2022.0097 35639360 PMC9918348

[16] Wittman SR, Martin JM, Mehrotra A, Ray KN. Antibiotic receipt during outpatient visits for COVID-19 in the US, from 2020 to 2022. JAMA Health Forum. 2023;4(2):e225429. doi:10.1001/jamahealthforum.2022.5429 36800196 PMC9938423

[17] American Society of Health System Pharmacists (ASHP). AHFS pharmacologic-therapeutic classification system. Accessed November 11, 2023. https://www.ashp.org/products-and-services/database-licensing-and-integration/ahfs-therapeutic-classification

[18] Foster CB, Martinez KA, Sabella C, Weaver GP, Rothberg MB. Patient satisfaction and antibiotic prescribing for respiratory infections by telemedicine. Pediatrics. 2019;144(3):e20190844. doi:10.1542/peds.2019-0844 31371464

[19] Shi Z, Barnett ML, Jena AB, Ray KN, Fox KP, Mehrotra A. Association of a clinician’s antibiotic-prescribing rate with patients’ future likelihood of seeking care and receipt of antibiotics. Clin Infect Dis. 2021;73(7):e1672-e1679. doi:10.1093/cid/ciaa1173 32777032 PMC8492129

[20] Ralston SL, Lieberthal AS, Meissner HC, ; American Academy of Pediatrics. Clinical practice guideline: the diagnosis, management, and prevention of bronchiolitis. Pediatrics. 2014;134(5):e1474-e1502. doi:10.1542/peds.2014-2742 25349312

[21] Shulman ST, Bisno AL, Clegg HW, ; Infectious Diseases Society of America. Clinical practice guideline for the diagnosis and management of group A streptococcal pharyngitis: 2012 update by the Infectious Diseases Society of America. Clin Infect Dis. 2012;55(10):e86-e102. doi:10.1093/cid/cis629 22965026 PMC7108032

[22] Lieberthal AS, Carroll AE, Chonmaitree T, . The diagnosis and management of acute otitis media. Pediatrics. 2013;131(3):e964-e999. doi:10.1542/peds.2012-3488 23439909

[23] Wald ER, Applegate KE, Bordley C, ; American Academy of Pediatrics. Clinical practice guideline for the diagnosis and management of acute bacterial sinusitis in children aged 1 to 18 years. Pediatrics. 2013;132(1):e262-e280. doi:10.1542/peds.2013-1071 23796742

[24] Iacus SM, King G, Porro G. Causal inference without balance checking: coarsened exact matching. Polit Anal. 2017;20(1):1-24. doi:10.1093/pan/mpr013

[25] Berta P, Bossi M, Verzillo S. %CEM: a SAS macro to perform coarsened exact matching. J Stat Comput Simul. 2017;87(2):227-238. doi:10.1080/00949655.2016.1203433

[26] Simon TD, Haaland W, Hawley K, Lambka K, Mangione-Smith R. Development and validation of the pediatric medical complexity algorithm (PMCA) version 3.0. Acad Pediatr. 2018;18(5):577-580. doi:10.1016/j.acap.2018.02.010 29496546 PMC6035108

[27] US Department of Agriculture Economic Research Service. Rural-urban commuting area codes. Accessed June 20, 2023. https://www.ers.usda.gov/data-products/rural-urban-commuting-area-codes/

[28] Brewster RCL, Khazanchi R, Butler A, O’Meara D, Bagchi D, Michelson KA. The 2022 to 2023 amoxicillin shortage and acute otitis media treatment. Pediatrics. 2023;152(3):e2023062482. 10.1542/peds.2023-062482 PMC10895544 37555262

[29] Martinez KA, Rood M, Rothberg MB. Coding bias in respiratory tract infections may obscure inappropriate antibiotic Use. J Gen Intern Med. 2019;34(6):806-808. doi:10.1007/s11606-018-4823-x 30652274 PMC6544729

[30] Mangione-Smith R, McGlynn EA, Elliott MN, Krogstad P, Brook RH. The relationship between perceived parental expectations and pediatrician antimicrobial prescribing behavior. Pediatrics. 1999;103(4 Pt 1):711-718. doi:10.1542/peds.103.4.711 10103291

[31] Martinez KA, Rood M, Jhangiani N, Kou L, Boissy A, Rothberg MB. Association between antibiotic prescribing for respiratory tract infections and patient satisfaction in direct-to-consumer telemedicine. JAMA Intern Med. 2018;178(11):1558-1560. doi:10.1001/jamainternmed.2018.4318 30285050 PMC6584324

[32] Martinez KA, Rood M, Jhangiani N, Boissy A, Rothberg MB. Antibiotic prescribing for respiratory tract infections and encounter length: an observational study of telemedicine. Ann Intern Med. 2019;170(4):275-277. doi:10.7326/M18-2042 30285078

[33] Cabral C, Lucas PJ, Ingram J, Hay AD, Horwood J. “It’s safer to …” parent consulting and clinician antibiotic prescribing decisions for children with respiratory tract infections: an analysis across four qualitative studies. Soc Sci Med. 2015;136-137:156-164. doi:10.1016/j.socscimed.2015.05.027 26004209

[34] Stenehjem E, Wallin A, Willis P, . Implementation of an antibiotic stewardship initiative in a large urgent care network. JAMA Netw Open. 2023;6(5):e2313011. doi:10.1001/jamanetworkopen.2023.13011 37166794 PMC10176123

[35] Du Yan L, Dean K, Park D, . Education vs clinician feedback on antibiotic prescriptions for acute respiratory infections in telemedicine: a randomized controlled trial. J Gen Intern Med. 2021;36(2):305-312. doi:10.1007/s11606-020-06134-0 32845446 PMC7878643

[36] Dutcher L, Degnan K, Adu-Gyamfi AB, . Improving outpatient antibiotic prescribing for respiratory tract infections in primary care: a stepped-wedge cluster randomized trial. Clin Infect Dis. 2022;74(6):947-956. doi:10.1093/cid/ciab602 34212177 PMC9630878

[37] Li LX, Szymczak JE, Keller SC. Antibiotic stewardship in direct-to-consumer telemedicine: translating interventions into the virtual realm. J Antimicrob Chemother. 2021;77(1):13-15. doi:10.1093/jac/dkab371 34618026

[38] Sanchez GV, Kabbani S, Tsay SV, . Antibiotic stewardship in outpatient telemedicine: adapting centers for disease control and prevention core elements to optimize antibiotic use. Telemed J E Health. Published online October 19, 2023. doi:10.1089/tmj.2023.0229 37856146

[39] National Committee for Quality Assurance (NCQA). Avoidance of antibiotic treatment for acute bronchitis/bronchiolitis (AAB). November 15, 2023. Accessed November 15, 2023. https://www.ncqa.org/hedis/measures/avoidance-of-antibiotic-treatment-for-acute-bronchitis-bronchiolitis/

[40] National Committee for Quality Assurance (NCQA). Appropriate treatment for upper respiratory infection (URI). Accessed November 15, 2023. https://www.ncqa.org/hedis/measures/appropriate-treatment-for-upper-respiratory-infection/

[41] Melville BL, Musser T, Fishman E, Rainis D, Byron SC. Developing a quality measure to assess use of antibiotic medications for respiratory conditions. Antimicrob Steward Healthc Epidemiol. 2023;3(1):e13. doi:10.1017/ash.2022.328 36714286 PMC9879865

[42] National Committee for Quality Assurance (NCQA). Antibiotic utilization for respiratory conditions (AXR). Accessed November 15, 2023. https://www.ncqa.org/hedis/measures/antibiotic-utilization/

[43] Ray KN, Keller D. Telehealth and pediatric care: policy to optimize access, outcomes, and equity. Pediatr Res. 2022;92(6):1496-1499. doi:10.1038/s41390-022-02306-2 36114243 PMC9483346

[44] Mehrotra A, Uscher-Pines L. Informing the debate about telemedicine reimbursement—what do we need to know? N Engl J Med. 2022;387(20):1821-1823. doi:10.1056/NEJMp2210790 36373824

[45] NCQA. Appendix 6: Proposed PCMH/PCSP telehealth distinction requirements. Accessed February 22, 2024. https://www.ncqa.org/wp-content/uploads/2020/11/20201117_Appendix_6_PCMHPCSP_Telehealth_Distinction_Requirements.pdf

[46] Powers BW, Drzayich Antol D, Zhao Y, . Association between primary care payment model and telemedicine use for medicare advantage enrollees during the COVID-19 pandemic. JAMA Health Forum. 2021;2(7):e211597. doi:10.1001/jamahealthforum.2021.1597 35977206 PMC8796918

