# Supplementary material for: Antibiotic Receipt for Pediatric Telemedicine Visits With Primary Care vs Direct-to-Consumer Vendors
Source: JAMA Netw Open. 2024 Mar 14;7(3):e242359. doi: 10.1001/jamanetworkopen.2024.2359 (PMC10940962; doi:10.1001/jamanetworkopen.2024.2359)
Supplement: Supplement 1. — eMethods. eResults. eTable 1. ICD-10 codes for diagnosis categories eTable 2. Visit counts for provider identifier codes identified as PCP telemedicine, DTC telemedicine, and excluded telemedicine providers eTable 3. Guideline-concordant antibiotic management by ARTI diagnosis eTable 4. ARTI visits and outcomes stratified by non-metropolitan and metropolitan child residence eTable 5. ARTI visits and outcomes, excluding visits with the presence of audio-only telemedicine codes eTable 6. Demographics and diagnoses of unmatched and matched index visits in sensitivity analysis eTable 7. ARTI visits and outcomes, “rigid” sensitivity analysis with index visits matched on diagnosis eTable 8. ARTI visits and outcomes, “relaxed” sensitivity analysis including symptom-only visits eTable 9. ARTI visits and outcomes, sensitivity analysis assigning visits with “excluded providers” to the DTC telemedicine group eTable 10. ARTI visits and outcomes, sensitivity analysis assigning visits with “excluded providers” to the PCP telemedicine group [file jamanetwopen-e242359-s001.pdf]

Supplementary Online Content

Wittman SR, Hoberman A, Mehrotra A, Sabik L, Yabes JG, Ray KN. Antibiotic receipt for pediatric telemedicine visits with primary care vs direct-to-consumer vendors *JAMA Netw Open*. 2024;7(3):e242359. doi:10.1001/jamanetworkopen.2024.2359

eMethods.

eResults.

eTable 1. ICD-10 codes for diagnosis categories

eTable 2. Visit counts for provider identifier codes identified as PCP telemedicine, DTC telemedicine, and excluded providers

eTable 3. Guideline-concordant antibiotic management by ARTI diagnosis

eTable 4. ARTI visits and outcomes stratified by non-metropolitan and metropolitan child residence

eTable 5. ARTI visits and outcomes, excluding visits with the presence of audio-only telemedicine codes

eTable 6. Demographics and diagnoses of unmatched and matched index visits in sensitivity analysis

eTable 7. ARTI visits and outcomes, “rigid” sensitivity analysis with index visits matched on diagnosis

eTable 8. ARTI visits and outcomes, “relaxed” sensitivity analysis including symptom-only visits

eTable 9. ARTI visits and outcomes, sensitivity analysis assigning visits with “excluded providers” to the DTC telemedicine group

eTable 10. ARTI visits and outcomes, sensitivity analysis assigning visits with “excluded providers” to the PCP telemedicine group

eReferences.

This supplementary material has been provided by the authors to give readers additional information about their work.

© 2024 Wittman SR et al. *JAMA Network Open*.

eMethods

Episodes of Care

An episode of care consists of an index ARTI visit (i.e., an ARTI visit which had no ARTI visit occurring in the prior 21 days) and any follow-up ARTI visits (which occurred within 14 days of a prior index ARTI visit) using methods consistent with prior studies.[1,2] We excluded 4% of episodes due to the child not having at least one month of continuous medical and pharmaceutical coverage at the time of visit (necessary to ensure capture outcomes) or lack of key demographic data (required for matching). We then excluded ARTI episodes where the index visit included an additional diagnosis which could affect antibiotic prescribing (e.g., cellulitis, urinary tract infection) using the list developed by Kronman *et al* (2% of episodes).[3] Because of our focus on antibiotic receipt following an initial outpatient visit at specific sites, we excluded episodes where patients visited multiple sites of care on the first day of the episode (1.0%) and where patients were hospitalized within 1 day of the first day of the episode (0.2%). Finally, because of unique considerations around SARS-CoV-2 during the COVID-19 pandemic,[2] we excluded episodes with a COVID-19 diagnosis (U07.1) on the index visit from this analysis (6%) (Figure 1).

Identifying Telemedicine Visits

Telemedicine visits were identified by CPT modifiers FQ, GT, GQ, 93 or 95; place of service code 2 or 10; or CPT codes 99441-99443.

Of note, we assessed indicators of audio-only versus audio-video visits for additional possible analysis. We found 5.2% of identified telemedicine visits had audio-only indicators (CPT codes 99441-99443 or CPT modifiers 93 or FQ), with this percentage varied by state from 0 to 27%. The wide state variation in presence of these indicators and low levels overall suggest that these indicators are likely specific but not sensitive for audio-only encounters. Given this, we did not match on the presence or absence of these audio-only codes in our main analysis. As an additional analysis, we did repeat our analysis after excluding visits with audio-only codes, which is presented as **eTable 5**.

© 2024 Wittman SR et al. *JAMA Network Open*.

eResults

Sensitivity Analyses
In our more rigid treatment of diagnosis, 12,929 PCP telemedicine index visits were matched to 13,123 DTC telemedicine index visits (**eTable 6**). Among this set of matched visits, the percent of visits receiving antibiotics during the initial visit was lower for PCP telemedicine index visits (27.5%) compared to DTC telemedicine visits (30.2%, RR: 0.91, 95% CI 0.87-0.96, **eTable 7**).

In this analysis, diagnoses were matched and therefore could not be examined as an outcome. Among matched visits receiving a streptococcal pharyngitis diagnosis, we found that streptococcal testing was billed within 2 days more often for PCP telemedicine visits (67.4%) than for DTC telemedicine visits (1.8%).

As in the main analysis, PCP telemedicine index visits were not significantly different in likelihood of receiving guideline-concordant antibiotic management compared to DTC telemedicine index visits (19.8 vs. 20.1%, RR: 0.98, 95% CI: 0.93-1.05).

Also consistent with the main analysis, PCP telemedicine index visits had lower rates of follow-up ARTI visits and antibiotics. For example, PCP telemedicine had fewer follow-up visits in the ensuring 1-2 days (5.1%) compared to DTC telemedicine visits (8.8%, RR: 0.58, 95% CI 0.51-0.66).

In our more relaxed treatment of diagnosis, 15,233 PCP telemedicine index visits were matched to 15,450 DTC telemedicine visits (**eTable 6**). Among this set of matched visits, the percent of visits receiving antibiotics during the initial visit was lower for PCP telemedicine index visits (27.7%) compared to DTC telemedicine visits (36.3%, RR: 0.76, 95% CI 0.73-0.80, **eTable 8**).

PCP telemedicine index visits were also less likely to result in a diagnosis where antibiotics may be appropriate (17.6% of PCP telemedicine visits) compared to DTC telemedicine index visits (26.8%, RR: 0.66, 95% CI 0.62-0.70).

PCP telemedicine index visits were not significantly different in likelihood of receiving guideline-concordant antibiotic management compared to DTC telemedicine index visits (19.7 vs. 20.1%, RR: 0.98, 95% CI: 0.92-1.04).

As in main analysis, PCP telemedicine index visits had lower rates of follow-up ARTI visits and antibiotics. For example, PCP telemedicine had fewer follow-up visits in the ensuring 1-2 days (6.3%) compared to DTC telemedicine visits (9.7%, RR: 0.65, 95% CI 0.59-0.72).

© 2024 Wittman SR et al. *JAMA Network Open*.

eTable 1. ICD-10 codes for diagnosis categories

ARTI diagnoses that may warrant antibiotics	ICD10 Codes
Streptococcal pharyngitis	J02.0, J03.00, and/or A38.n
Acute Otitis Media (AOM)	H66.00n, H66.01n, H66.1n, H66.2n, H66.3n, H66.4n, H66.9n, and/or H67.n
Sinusitis	J01.0n, J01.1n, J01.2n, J01.3n, J01.4n, J01.8n, and/or J01.9n
ARTI diagnoses that do not warrant antibiotics	
Bronchiolitis/Bronchiolitis	J20.3, J20.4, J20.5, J20.6, J20.7, J20.8, J20.9, J21.n, and/or J40
Other Viral	J00, J02.8, J02.9, J03.8n, J03.9n, J06.n, and/or H65.n
Influenza	J10.1 and/or J11.1
ARTI symptoms* (included only in 2nd sensitivity analysis)	
ARTI symptoms	R05.1n, R05.2n, R05.8n, R05.9n, R06.0n, R06.1n, R06.2n, R06.7n, R06.82, R07.0n, R07.81, R09.3n, R09.81, R09.82, R50.9n, and/or R06.89<br>AND<br>No non-RXX code (i.e., only symptoms entered as diagnoses for the visit)

*ARTI symptoms were used to identify visits to include in 2nd sensitivity analysis which incorporated visits with symptoms potentially consistent with ARTIs (e.g., rhinorrhea, cough) if no actual diagnosis was entered for that encounter. Visits with ARTI symptom codes but no ARTI diagnosis codes were not included in the main analysis.

© 2024 Wittman SR et al. *JAMA Network Open*.

eTable 2. Visit counts for provider identifier codes identified as PCP telemedicine, DTC telemedicine, and excluded telemedicine providers

	**PCP Telemedicine**	**DTC Telemedicine**	**Excluded**
Provider Identifier Codes, n	6,994	6	351
Patients per provider code, range	20-293,851	17,058-403,832	20-6,217
Percent telemedicine, range	0.1-49.6%	97.4-100.0%	50.0%-100.0%
Number of pediatric ARTI telemedicine index visits	15,117 (45.6%)	15,519 (46.8%)	2,531 (7.6%)

© 2024 Wittman SR et al. *JAMA Network Open*.

eTable 3. Guideline-concordant antibiotic management by ARTI diagnosis

**Diagnosis**	**Guideline Concordant Management**	**Non-Guideline-Concordant Management**
Streptococcal Pharyngitis	Receipt of Amoxicillin or<br>Receipt of Penicillin	Receipt of any other systemic antibiotic<br>No receipt of antibiotics
Acute Otitis Media	Receipt of Amoxicillin or<br>Receipt of Amoxicillin/clavulanic acid or<br>No receipt of antibiotics	Receipt of any other systemic antibiotic
Sinusitis	Receipt of Amoxicillin or<br>Receipt of Amoxicillin/clavulanic acid or<br>No receipt of antibiotics	Receipt of any other systemic antibiotic
Influenza	No receipt of antibiotics	Receipt of any systemic antibiotic
Bronchitis/Bronchiolitis	No receipt of antibiotics	Receipt of any systemic antibiotic
Other Viral URIs	No receipt of antibiotics	Receipt of any systemic antibiotic
Symptom-only visits	No receipt of antibiotics	Receipt of any systemic antibiotic

© 2024 Wittman SR et al. *JAMA Network Open*.

eTable 4. ARTI visits and outcomes stratified by non-metropolitan and metropolitan child residence

	PCP Telemedicine	DTC Telemedicine	
	Weighted % (95% CI)	Weighted % (95% CI)	RR (95% CI)
Matched non-metropolitan index visits, n	972	915	
Received antibiotics	41.6 (38.2-44.9)	50.2 (44.8-55.5)	**0.83 (0.72-0.95)**
Received antibiotics-may-be-indicated diagnoses	26.2 (23.4-29.1)	36.3 (31.0-41.7)	**0.72 (0.60-0.87)**
Received antibiotic management not concordant with given diagnosis	31.4 (28.3-34.5)	29.7 (24.7-34.8)	1.06 (0.87-1.29)
Matched metropolitan index visits, n	13,230	13,712	
Received antibiotics	28.0 (27.2-28.8)	36.3 (35.0-37.5)	**0.77 (0.74-0.81)**
Received antibiotics-may-be-indicated diagnoses	18.5 (17.8-19.2)	27.9 (26.7-29.1)	**0.66 (0.63-0.70)**
Received antibiotic management not concordant with given diagnosis	19.4 (18.7-20.1)	19.4 (18.5-20.3)	1.00 (0.94-1.06)

Legend: Results of analysis after stratifying matched visits by non-metropolitan (having a micropolitan, small town, or rural RUCA code) vs metropolitan (having a metropolitan RUCA code) child residence.

© 2024 Wittman SR et al. *JAMA Network Open*.

eTable 5. ARTI visits and outcomes, excluding visits with the presence of audio-only telemedicine codes

	PCP Telemedicine	DTC Telemedicine	
	Weighted % (95% CI)	Weighted % (95% CI)	RR (95% CI)
Matched audio-video index visits, n	13,372	14,225	
Received antibiotics	28.8 (28.0-29.6)	37.2 (35.9-38.4)	**0.77 (0.74-0.81)**
Received antibiotics-may-be-indicated diagnoses	19.2 (18.5-19.9)	28.3 (27.1-29.5)	**0.68 (0.64-0.72)**
Received antibiotic management not concordant with given diagnosis	20.1 (19.4-20.8)	19.9 (19.0-20.9)	1.01 (0.95-1.07)

Legend: Results of analysis excluding visits with audio-only codes (6.1% of unmatched PCP telemedicine index visits and 2.4% of unmatched DTC telemedicine index visits) prior to matching. Audio-only codes were determined by the presence of CPT codes 99441-99443, CPT modifiers 93 or FQ, or place or service code 10.

© 2024 Wittman SR et al. *JAMA Network Open*.

eTable 6. Demographics and diagnoses of unmatched and matched ARTI index visits in sensitivity analysis

	Unmatched		Rigid Treatment of Diagnosis: Matched on sociodemographics and diagnosis		Relaxed Treatment of Diagnosis: Including symptom-only visits, matched on sociodemographics	
	PCP TM	DTC TM	PCP TM	DTC TM	PCP TM	DTC TM
N, unweighted	15,117	15,519	12,929	13,123	15,233	15,450
	Number (percent)		*Weighted percent*		*Weighted percent*	
Child age						
– 0-1	1471 (10%)	527 (3%)	8%	8%	9%	9%
– 2-5	3990 (26%)	3325 (21%)	26%	26%	27%	27%
– 6-11	4804 (32%)	5921 (28%)	33%	33%	32%	32%
– 12-17	4852 (32%)	5746 (37%)	33%	33%	32%	32%
Child sex						
– M	7611 (50%)	7749 (50%)	51%	51%	51%	51%
– F	7501 (50%)	7758 (50%)	49%	49%	49%	49%
– U	<11 (<0.1%)	12 (<0.1%)	0%	0%	0%	0%
Medical complexity						
- Non-Chronic	10022 (66%)	11086 (71%)	70%	70%	68%	68%
- Non-complex Chronic	3397 (22%)	3112 (20%)	21%	21%	22%	22%
- Complex Chronic	1698 (11%)	1321 (9%)	9%	9%	10%	10%
Census Region						
- Northeast	1649 (11%)	713 (5%)	10%	10%	10%	10%
- South	7115 (47%)	9052 (58%)	50%	50%	48%	48%
- Midwest	2702 (18%)	2896 (19%)	17%	17%	18%	18%
- West	3651 (24%)	2858 (18%)	24%	24%	24%	24%
Rural urban						
- Metropolitan	13602 (90%)	14011 (90%)	95%	95%	93%	93%
- Micropolitan	788 (5%)	862 (6%)	3%	3%	4%	4%
- Small Town	493 (3%)	424 (3%)	1%	1%	2%	2%
- Rural	234 (2%)	222 (1%)	0.4%	0.4%	1%	1%
Antibiotics-may-be-indicated diagnoses	2918 (19%)	4789 (30%)	17%	17%	18%	27%
- Streptococcal pharyngitis	768 (5%)	1123 (7%)	5%	5%	5%	6%
- Acute otitis media	645 (4%)	973 (6%)	3%	3%	4%	7%
- Sinusitis	1505 (10%)	2693 (17%)	9%	9%	9%	15%
-						
Antibiotics-not-indicated diagnoses	12199 (81%)	10730 (70%)	83%	83%	82%	73%
– Influenza	765 (5%)	571 (4%)	4%	4%	5%	3%
– Bronchitis/Bronchiolitis	606 (4%)	535 (4%)	3%	3%	4%	3%
– Other Viral	10828 (72%)	9624 (62%)	77%	77%	67%	60%
– Symptom-only	-	-	-	-	7%	6%

© 2024 Wittman SR et al. *JAMA Network Open*.

eTable 7. ARTI visits and outcomes, "rigid" sensitivity analysis with index visits matched on diagnosis

	PCP Telemedicine	DTC Telemedicine	
	Weighted % (95% CI)	Weighted % (95% CI)	RR (95% CI)
Matched index visits, n<br>- Antibiotic-may-be-indicated diagnoses<br>- Antibiotic-not-indicated diagnoses	12,929<br>17.1<br>82.9	13,123<br>17.1<br>82.9	
**Index Visit**			
Received antibiotics	27.5 (26.7-28.3)	30.2 (29.1-31.4)	**0.91 (0.87-0.96)**
Received antibiotic management not concordant with given diagnosis<br>-Antibiotic indicated but not prescribed<br>-Antibiotic selection not guideline concordant<br>-Antibiotic not indicated but prescribed	19.8 (19.1-20.5)<br>0.9 (0.7-1.1)<br>5.2 (4.8-5.6)<br>13.8 (13.1-14.4)	20.1 (19.1-21.1)<br>0.7 (0.5-0.8)<br>2.8 (2.5-3.1)<br>16.7 (15.7-17.6)	0.98 (0.93-1.05)<br>**1.37 (1.02-1.83)**<br>**1.85 (1.61-2.12)**<br>**0.82 (0.77-0.89)**
**Follow-up Care within 1-2 days**			
Follow-up ARTI Visit within 1-2 days	5.1 (4.7-5.5)	8.8 (7.9-9.6)	**0.58 (0.51-0.66)**
Antibiotics filled after ARTI visit within 1-2 days	1.7 (1.5-2.0)	3.5 (3.0-4.0)	**0.50 (0.41-0.61)**
**Follow-up Care within 3-14 days**			
Follow-up ARTI Visit within 3-14 days	8.2 (7.7-8.7)	9.8 (8.9-10.6)	**0.84 (0.76-0.93)**
Antibiotics filled after ARTI visit within 3-14 days	3.2 (2.9-3.5)	4.9 (4.3-5.5)	**0.65 (0.55-0.76)**

Legend: Results of sensitivity analysis in which visits were matched on sociodemographic characteristics as well as ARTI diagnosis.

© 2024 Wittman SR et al. *JAMA Network Open*.

eTable 8. ARTI visits and outcomes, “relaxed” sensitivity analysis including symptom-only visits

	PCP Telemedicine	DTC Telemedicine	
	Weighted % (95% CI)	Weighted % (95% CI)	RR (95% CI)
Matched index visits, n	15,233	15,450	
**Index visit**			
Received antibiotics	27.7 (27.0-28.5)	36.3 (35.1-37.5)	**0.76 (0.73-0.80)**
Received antibiotics-may-be-indicated diagnoses	17.6 (17.0-18.3)	26.8 (25.6-27.9)	**0.66 (0.62-0.70)**
- Streptococcal Pharyngitis	4.7 (4.4-5.1)	5.7 (5.2-6.2)	**0.83 (0.74-0.93)**
- Acute Otitis Media	3.8 (3.5-4.1)	6.5 (5.6-7.4)	**0.58 (0.49-0.68)**
- Sinusitis	9.1 (8.7-9.6)	14.5 (13.7-15.4)	**0.63 (0.58-0.68)**
Received antibiotics-not-indicated diagnoses	82.4 (81.8-83.0)	73.2 (72.1-74.4)	**1.12 (1.11-1.14)**
- Influenza	4.7 (4.3-5.0)	3.1 (2.7-3.6)	**1.49 (1.28-1.74)**
- Bronchitis/Bronchiolitis	3.7 (3.4-4.0)	3.3 (2.9-3.7)	1.12 (0.96-1.31)
- Other Viral	66.6 (65.9-67.4)	60.4 (59.1-61.6)	**1.10 (1.08-1.13)**
- Symptom-only	7.4 (7.0-7.8)	6.4 (5.8-7.1)	**1.15 (1.03-1.29)**
Received antibiotic management not concordant with given diagnosis	19.7 (19.0-20.3)	20.1 (19.2-21.1)	0.98 (0.92-1.04)
- Antibiotic indicated but not prescribed	0.9 (0.8-1.1)	0.8 (0.6-0.9)	1.20 (0.91-1.57)
- Antibiotic selection not guideline concordant	5.2 (4.8-5.6)	4.5 (4.1-5.0)	**1.15 (1.01-1.30)**
- Antibiotic not indicated but prescribed	13.5 (13.0-14.1)	14.8 (14.0-15.6)	**0.91 (0.85-0.98)**
**Follow-up care within 1-2 days**			
Follow-up ARTI Visit within 1-2 days	6.3 (5.9-6.7)	9.7 (8.9-10.5)	**0.65 (0.59-0.72)**
Antibiotics filled after ARTI visit within 1-2 days	2.1 (1.9-2.3)	3.7 (3.2-4.2)	**0.57 (0.48-0.67)**
**Follow-up care within 3-14 days**			
Follow-up ARTI Visit within 3-14 days	10.6 (10.1-11.1)	11.8 (11.0-12.6)	**0.89 (0.82-0.97)**
Antibiotics filled after ARTI visit within 3-14 days	3.8 (3.5-4.1)	5.5 (5.0-6.1)	**0.68 (0.60-0.78)**

Legend: Results of sensitivity analysis in which symptom-only visits were included and visits were matched on sociodemographic characteristics.

© 2024 Wittman SR et al. *JAMA Network Open*.

eTable 9. ARTI visits and outcomes, sensitivity analysis assigning visits with "excluded providers" to the DTC telemedicine group

	PCP Telemedicine	DTC Telemedicine	
	Weighted % (95% CI)	Weighted % (95% CI)	RR (95% CI)
Matched index visits, n	14,293	17,110	
**Index visit**			
Received antibiotics	28.9 (28.2-29.7)	36.1 (35.0-37.2)	**0.80 (0.77-0.83)**
Received antibiotics-may-be-indicated diagnoses	19.1 (18.4-19.7)	27.4 (26.4-28.4)	**0.70 (0.66-0.73)**
Received antibiotic management not concordant with given diagnosis	19.9 (19.0-20.7)	20.2 (19.5-20.9)	1.02 (0.97-1.08)

Legend: Results of sensitivity analysis in which visits to telemedicine provider identifier codes excluded from the main analysis were included in the DTC telemedicine category prior to matching. Thus, in this analysis, the 6994 provider identifier codes identified as having <50% of visits conducted via telemedicine were categorized as "PCP telemedicine", while 357 provider identifier codes with ≥50% of visits conducted via telemedicine were categorized as "DTC telemedicine."

© 2024 Wittman SR et al. *JAMA Network Open*.

eTable 10. ARTI visits and outcomes, sensitivity analysis assigning visits with "excluded providers" to the PCP telemedicine group

	PCP Telemedicine	DTC Telemedicine	
	Weighted % (95% CI)	Weighted % (95% CI)	RR (95% CI)
Matched index visits, n	16,666	14,692	
**Index visit**			
Received antibiotics	28.4 (27.7-29.1)	37.1 (35.9-38.3)	**0.77 (0.74-0.80)**
Received antibiotics-may-be-indicated diagnoses	19.2 (18.5-19.8)	28.3 (27.1-29.4)	**0.68 (0.64-0.71)**
Received antibiotic management not concordant with given diagnosis	19.2 (18.6-19.8)	20.1 (19.2-21.0)	0.96 (0.90-1.01)

Legend: Results of sensitivity analysis in which visits to telemedicine provider identifier codes excluded from the main analysis were included in the PCP telemedicine category prior to matching. Thus, in this analysis, the 6-provider identifier code cluster with virtually all telemedicine use (97.4-100%) and ≥10,000 visits were categorized as "DTC telemedicine" and compared to the remaining 7,345 provider identifier codes which were categorized as "PCP telemedicine".

© 2024 Wittman SR et al. *JAMA Network Open*.

eReferences

1. Wittman SR, Yabes JG, Sabik LM, Kahn JM, Ray KN. Patient and Family Factors Associated with Use of Telemedicine Visits for Pediatric Acute Respiratory Tract Infections, 2018-2019. *Telemed J E Health.* 2023;29(1):127-136.
2. Wittman SR, Martin JM, Mehrotra A, Ray KN. Antibiotic Receipt During Outpatient Visits for COVID-19 in the US, From 2020 to 2022. *JAMA Health Forum.* 2023;4(2):e225429.
3. Kronman MP, Gerber JS, Grundmeier RW, et al. Reducing Antibiotic Prescribing in Primary Care for Respiratory Illness. *Pediatrics.* 2020;146(3).

© 2024 Wittman SR et al. *JAMA Network Open*.
