# Supplementary material for: Antibiotic Receipt for Pediatric Telemedicine Visits With Primary Care vs Direct-to-Consumer Vendors
Source: JAMA Netw Open. 2024 Mar 14;7(3):e242359. doi: 10.1001/jamanetworkopen.2024.2359 (PMC10940962; doi:10.1001/jamanetworkopen.2024.2359)
Supplement: Supplement 2. — Data Sharing Statement [file jamanetwopen-e242359-s002.pdf]

Data Sharing Statement

Data

Data available: No

Additional Information

Explanation for why data not available: Access to these data is managed through Optum Labs Data Warehouse.
